# Monthly Summary

**Published:** 1873-09

**Authors:** 


### MONTHLY SUMMARY.

---

*Chloral as an Application in Fetid Ulcers.*—MM. Dusardin-Beaumetz and Hirine communicated at a recent meeting of the Paris Medical Hospital Society the result of the investigations they have been making into the "Anti Putrid and Anti-Fermentiscible Properties of Solutions of Hydrate of Chloral and their Therapeutical Applications." Their attention was first drawn to the subject by the remarkable success which attended the application of a solution of chloral diluted by 100 parts of water to a vast eschar of the buttocks which occured during an attack of typhoid. Since then they have employed the solution in the treatment of various wounds in bad condition, and in suppuration occuring in closed cavities.

With respect to the therapeutical applications, the authors observe that the action of diluted chloral on wounds of a bad nature has been known for some time; Burggraeve having indicated it as one of the best applications. In Italy it has acquired a great reputation, and Dr. Francisco reports the great benefit he has derived from its use in a concentrated from (5 parts to 20) in inveterate ulcers The authors have also employed it in numerous cases of gangrenous wounds and wounds of a bad character, but they have in the great majority of these confined themselves to solutions of 1-50 or 1-100. Several of these cases are published in the *Union Medicale* (May 27 and 29;) and the authors believe themselves authorized to recommend the use of chloral under these circumstances. So also, as it prevents decomposition of the urine, it may render great service in affections of the urinary organs.—*Med. Times and Gazette.*

---

*Death from Methylene Ether.*—Mr. Lawson Tait reports (*Med. Times and Gaz.*, July 5, 1873) the case of S. S., aged 62, admitted to the hospital for women, for a large multilocular ovarian tumour. She was placed under the influence of methylene ether and took it readily and quietly; after five drachms had been administered she seemed so perfectly unconscious that in a few seconds the operation would have been begun. Suddenly she seemed to be coming out of the anæsthesia, the urine passed out of her bladder, and the eyes opened. Mr. Tait noticed the pupils to be extremely dilated, and the pulse suddenly ceased. Some efforts at respiration were made, and a spasmodic effort as if the patient was about to vomit. Respiration then ceasing, it was carried on by Sylvester's method. Strong ammonia was applied under the nostrils, and a strong stimulant enema given. Other means, as rubbing the chest with a brush, dashing cold water over it, etc., were tried unavailingly. It was evident to the acting staff, who were all present, that failure of the heart was the initial part of the process of death.

The post-mortem examination was made within twenty-four hours, and revealed nothing to account for death.—*Med. News.*

---

*Cholera.*—Asiatic cholera has again visited the United States, and has made its appearance about two months earlier than it did in 1866. During the last epidemic of this fatal malady, I was the resident physician of the Cincinnati Hospital, and my opportunities for observing the disease in all its stages, and testing the efficacy of various remedies in its treatment, were by no means limited. In almost every case of cholera there is a premonitory diarrhœa, lasting from a few hours to several days, which, if properly treated, is readily cured. In time of cholera every movement of the bowels, after what is customary and natural, must be regarded as a tendency toward the disease, and after diarrhœa is established, the patient should go to bed and have a physician summoned immediately. The following remedy has been used by the writer in hundreds of cases of cholera, and it will arrest almost every case of the disease in the *first stage.*

Take of laudanum, spirit of camphor and tincture of ginger, each four fluid drachms; tincture capsicum and chloroform, each two fluid drachms; pure brandy, two fluid ounces. Mix. Dose for an adult, a teaspoonful in ice water after each movement of the bowels.

The above remedy will prove as effectual in the rice-water stage as in the first or prodromic stage.—*Druggists' Circular*

---

*Ingrowing Toe-Nails.*—A correspondent of the *British Medical Journal* takes the ground that no cutting operation is at all necessary for the complete and rapid cure of ingrowing toe-nail.

If a small, thin flat piece of silver plate be bent at one edge into a slight deep groove, and after the toe has been poulticed twenty four hours, slipped beneath the edge of the nail, so as to protect the flesh from its pressure, and the rest of the thin plate bent round the side and front of the toe, being kept in position with a small portion of resin plaster passed round the toe, a speedy and almost painless cure will take place; and the patient, after the first day, has the additional advantage of being able to walk. This method has been followed in numerous cases with uniform success.

Dr. Blower, of Liverpool, states, in the same journal, that he has, for the past twenty years employed compressed sponge successfully in the treatment of ingrowing nails. He renders the sponge compact by wetting and then tying it tightly, until it is thoroughly dry. A bit of the sponge, in size less than a grain of rice, is placed under the nail, and secured by strips of adhesive plaster. In this way the point of the nail is kept up from the toe until the surrounding soft parts are restored to their normal condition by appropriate means.—*Druggists' Circular.*

---

*Detection of the Substitution of Carbolic Acid for Creasote*—In the *Can. Pharm: Journal*, No. 12, vol. 5, there is a communication from Mr. Morrison, London, on the substitution of carbolic acid for creasote. He states that there is no good test for distinguishing between the two, but proposes the use of glycerine, in which carbolic acid is easily soluble, but creasote insoluble. A far better test is the alcoholic solution of perchloride iron [or tr. ferri perchlor. B. P.], which when added to an alcoholic solution of creasote, produces a "dark greenish-blue" color, but with an alcoholic solution of carbolic acid only a "light brown" coloration. By this test 1 part of creasote in 500 parts carbolic acid can be easily detected. But the adulteration of creasote by carbolic acid is more difficult to detect, but can be ascertained in the following way: Boil a few drops creasote with nitric acid [about 2 drs.] until red fumes are no longer evolved; this yields a solution, which, when neutralized with solution of caustic potash, gives *no precipitate*, the creasote forming oxalic acid. Carbolic acid, when treated in the same manner, is very violently acted on by nitric acid, and forms picric acid. [trinitro-phenylic acid] which when neutralized with solution of potassa, gives a "yellow crystalline" precipitate. One part of carbolic acid in 50 parts creasote can be readily detected in this way.—*Canada Phar. Journal.*

---

*Suppression of Perspiration.*—Socoloff gives an abstract of the results which follow varnishing the skin and suppression of the cutaneous secretion.

1. A few hours before the death of the animals so treated, clonic and tetanic spasms appear in various groups of muscles, while the temperature in the rectum sinks in a marked degree.

2. Enveloping the animals in wadding did not serve to raise the temperature or arrest the fatal result.

3 Respiration of oxygen proved ineffectual to resuscitate the animals.

4. In the stomach ulcers were observed, the result of deep extravasations.

5. Albumen appeared in the urine very soon after the skin was varnished.

6. In all cases a diffuse parenchymatous inflammation of the kidneys was observed—sometimes swelling of the cells, and sometimes fatty degeneration. This result was independent of the nature of the varnish used, whether turpentine varnish or gelatine or gum.

Lang [*Arch. d. Heilkunds*, xiii., pp, 277—287, 1872] investigates the cause of death when the skin has been varnished. In addition to other phenomena he found an hour or two after death "triple phosphate crystals" in various parts of the body, and some of the uriniferous tubules blocked with a finely granular dark mass. He thinks that the triple phosphate crystals are the result cf decomposition of urea, and that the cause of death is uræmia.—*Am. Journal of Med. Sciences.*

